# In vitro activity of rhinacanthin analogues against drug resistant *Plasmodium falciparum* isolates from Northeast Thailand

**DOI:** 10.1186/s12936-023-04532-3

**Published:** 2023-03-23

**Authors:** Suwanna Chaorattanakawee, Varakorn Kosaisavee, Watanyu Bunsermyos, Chaiyawat Aonsri, Witcha Imaram, Kanokon Suwannasin, Chanon Kunasol, Chatchadaporn Thamnurak, Nonlawat Boonyalai, David Saunders, Arjen M. Dondorp, Mathirut Mungthin, Mallika Imwong

**Affiliations:** 1 Department of Parasitology and Entomology, Faculty of Public Health, Mahidol University, Bangkok, Thailand; 2 Department of Pharmaceutical Chemistry, Faculty of Pharmacy, Mahidol University, Bangkok, 10400 Thailand; 3 Department of Chemistry and Center of Excellence for Innovation in Chemistry, Faculty of Science, Kasetsart University, Bangkok, Thailand; 4 Mahidol-Oxford Tropical Medicine Research Unit, Faculty of Tropical Medicine, Mahidol University, Bangkok, Thailand; 5 Department of Bacterial and Parasitic Diseases, Armed Forces Research Institute of Medical Science (AFRIMS), Bangkok, Thailand; 6 Uniformed Services University of the Health Sciences, Bethesda, MD USA; 7 Centre for Tropical Medicine and Global Health, Nuffield Department of Medicine, University of Oxford, Oxford, UK; 8 Department of Parasitology, Phramongkutklao College of Medicine, 317 Ratchawithi Road, Ratchathewi, Bangkok, 10400 Thailand; 9 Department of Molecular Tropical Medicine and Genetics, Faculty of Tropical Medicine, Mahidol University, Bangkok, Thailand

## Abstract

**Background:**

New anti-malarial drugs are needed urgently to address the increasing challenges of drug-resistant falciparum malaria. Two rhinacanthin analogues containing a naphthoquinone moiety resembling atovaquone showed promising in-vitro activity against a *P. falciparum* laboratory reference strain (K1). The anti-malarial activity of these 2 compounds was further evaluated for *P. falciparum* field isolates from an area of multi-drug resistance in Northeast Thailand.

**Methods:**

Using a pLDH enzyme-linked immunosorbent assay, four *P. falciparum* isolates from Northeast Thailand in 2018 were tested for in vitro sensitivity to the two synthetic rhinacanthin analogues 1 and 2 as well as established anti-malarials. Mutations in the *P. falciparum* cytochrome b gene, a marker for atovaquone (ATQ) resistance, were genotyped in all four field isolates as well as 100 other clinical isolates from the same area using PCR-artificial Restriction Fragment Length Polymorphisms. *Pfkelch13* mutations, a marker for artemisinin (ART) resistance, were also examined in all isolates.

**Results:**

The 50% inhibitory concentrations (IC_50_) of *P. falciparum* field isolates for rhinacanthin analogue 1 was 321.9–791.1 nM (median = 403.1 nM). Parasites were more sensitive to analogue 2: IC_50_ 48.6–63.3 nM (median = 52.2 nM). Similar results were obtained against *P. falciparum* reference laboratory strains 3D7 and W2. The ART-resistant IPC-5202 laboratory strain was more sensitive to these compounds with a median IC_50_ 45.9 and 3.3 nM for rhinacanthin analogues 1 and 2, respectively. The ATQ-resistant C2B laboratory strain showed high-grade resistance towards both compounds (IC_50_ > 15,000 nM), and there was a strong positive correlation between the IC_50_ values for these compounds and ATQ (r = 0.83–0.97, P < 0.001). There were no *P. falciparum* cytochrome b mutations observed in the field isolates, indicating that *P. falciparum* isolates from this area remained ATQ-sensitive. *Pfkelch13* mutations and the ring-stage survival assay confirmed that most isolates were resistant to ART.

**Conclusions:**

Two rhinacanthin analogues showed parasiticidal activity against multi-drug resistant *P. falciparum* isolates, although less potent than ATQ. Rhinacanthin analogue 2 was more potent than analogue 1, and can be a lead compound for further optimization as an anti-malarial in areas with multidrug resistance.

**Supplementary Information:**

The online version contains supplementary material available at 10.1186/s12936-023-04532-3.

## Background

The rapid spread of drug resistant-malaria is a global public health problem threatening efforts to control and eliminate malaria. Currently, artemisinin-based combination therapy (ACT) is the first-line treatment for uncomplicated falciparum malaria in all malaria endemic countries. However, the emergence and spread of parasite resistance to artemisinin and partner drugs in the Greater Mekong sub-region (GMS) have made malaria treatment less effective [1]. Artemisinin resistance first emerged in 2008 in western Cambodia along the Thai border, and later spread to Thailand, Laos, Vietnam, and Myanmar [2–4]. From 2008 to 2015, dihydroartemisinin-piperaquine (DHA-PPQ) was implemented in Cambodia and Thailand to replace artesunate-mefloquine (AS-MQ) due to increasing AS-MQ failure rates [5–7]. A rapid rise in PPQ resistance resulted in high rates of DHA-PPQ failure just a few years after implementation [1, 8–11], prompting a change in first-line treatment to AS-MQ in Cambodia, and to artesunate-pyronaridine in Northeastern Thailand and Vietnam [9, 12–15]. These increasing problems with anti-malarial drug resistance highlight the urgent need for the development of new anti-malarial drugs.

**Fig. 1:**
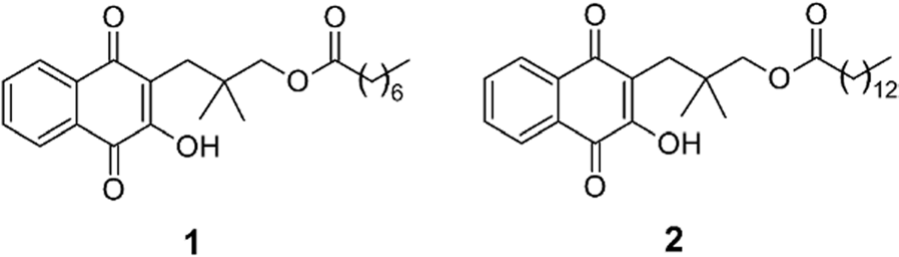
Chemical structure of rhinacanthin analogues 1 and 2

Rhinacanthins are quinone compounds belonging to the naphthoquinone group [16]. Their natural source is *Rhinacanthus nasutus*, a medicinal herb distributed throughout tropical and subtropical regions. *Rhinacanthus nasutus* is used as a traditional medicine to treat cancer, fungal and herpes virus infections, skin disease, and various other disorders [17]. The herb extract has shown cytotoxic potency against several carcinoma cell lines, as well as antifungal, antimicrobial, and antiviral activity [18–21]. Recently, two rhinacanthin analogues, designated **1** and **2**, containing a naphthoquinone moiety resembling atovaquone (ATQ) (Fig. 1) have been synthesized. Both have shown promising in vitro parasiticidal activity against *P. falciparum*, with no apparent toxicity in Vero cell lines [16]. Similar to ATQ, these compounds have potent inhibition against *P. falciparum* cytochrome bc1, but less effect on yeast cytochrome bc1, and no activity on rat cytochrome bc1. Anti-malarial activities have been tested to date only against *P. falciparum* K1 strains, but not against ATQ-resistant strains. This prompted to further evaluate the activity of these 2 compounds against *P. falciparum* ATQ-resistant laboratory strains (C2B) as well as multi-drug resistant *P. falciparum* field isolates from Ubon Ratchathani province in northeast Thailand. *Plasmodium falciparum* laboratory strains with different drug resistance profiles were tested in parallel. Compound activity against drug-resistant *P. falciparum* isolates and cross-susceptibility to ATQ were evaluated. *Plasmodium falciparum* isolates were also analyzed for presence of the genetic marker for ATQ resistance, the Y268S mutation in the cytochrome b gene.

## Methods

### Study sites and samples

In 2018, 4 *P. falciparum* isolates from Ubon Ratchathani province in northeast Thailand were collected at the time of diagnosis for in vitro drug/compound susceptibility testing. All samples were collected from patients with *P. falciparum* mono-infections visiting the Na Chaluai district malaria clinic on the Thai-Laos border, and close to the Cambodian border. Fresh patient blood samples (3 mL) were used for malaria culture and drug susceptibility testing. A 200 μL aliquot was used to evaluate cytochrome b mutations for atovaquone (ATQ) resistance and *Pfkelch13* mutations as markers for artemisinin (ART) resistance. Parasite isolates were then culture-adapted to perform the ring-stage assay to assess ART susceptibility and the piperaquine survival assay for PPQ susceptibility. *Plasmodium falciparum* DNA samples extracted from blood samples of 100 patients with uncomplicated falciparum malaria from various districts in Ubon Ratchathani province on the Thai-Laos and Thai-Cambodia border collected in 2014–2015 were also analyzed for cytochrome b and *Pfkelch13* mutations. The study was approved by the Ethical Review Committee for Human Research, Faculty of Public Health, Mahidol University, and the Ethical Committee for Research in Human Subjects, Ministry of Public Health, Thailand. All study subjects provided informed consent prior to participation.

In addition to field isolates, reference *P. falciparum* laboratory strains with different drug resistance profiles were tested for in vitro susceptibility in parallel. The laboratory strains W2/Indochina, 3D7/Africa, C2B/Thailand, and IPC-5202/Cambodia (Malaria Research & Reference Reagent Resource, Manassas, Vermont, USA) are resistant to chloroquine (CQ), mefloquine (MQ), ATQ, and ART, respectively.

### Synthesis of rhinacanthin analogues 1 and 2 and preparation of drug-coated plates

The synthesis of rhinacanthin analogues 1 and 2 was adapted from previous published methods by Kongkathip et al. [16]. Rhinacanthin analogues 1 and 2 were dissolved in absolute ethanol as a 1 mg/mL stock solution. CQ, MQ, quinine (QN), and ATQ were kindly provided by the World Wide Anti-malarial Resistance Network (WWARN). The drug stock solution (1 mg/mL) was prepared in 70% ethanol, except for ATQ, which was dissolved in absolute ethanol. The stock solutions of the compounds were subsequently diluted to appropriate working concentrations in sterile distilled water, and coated in 96 well plates as previously described [9]. Briefly, three-fold serial drug dilutions were used providing final drug concentrations ranging from 0.66 to 482 nM for MQ, 2.18 to 1596 nM for QN, 5.31 to 3877 nM for CQ, 0.38 to 273 nM for ATQ, 7.10 to 5175 nM for rhinacanthin analogue 1 and 2.91 to 2125 nM rhinacanthin analogue 2. In vitro drug sensitivity of the ATQ-resistant C2B strain used higher ATQ concentration, ranging from 374 to 272,580 nM. Drug and compound concentration were tested in singlet and duplicate, respectively. The culture plates were dried overnight and subsequently kept at 4 ˚C until used.

### Parasite susceptibility assay to rhinacanthin analogues 1 and 2 and standard anti-malarial drugs

For *P. falciparum* field isolates, blood samples were tested for in vitro susceptibility, following published methods [22]. Briefly, patient samples with a parasitaemia of up to 0.5% were adjusted to 1.5% haematocrit in RPMI 1640 medium supplemented with 10% human serum. Samples with a parasitaemia > 0.5% were diluted to obtain a 0.5% parasitaemia prior to adjusting to a 1.5% haematocrit. The cell-medium mixture (200 μL) was then transferred to dried drug-coated plates. Plates were placed in a candle jar and incubated at 37 °C for 72 h. A *Plasmodium* pan-species lactate dehydrogenase enzyme-linked immunosorbent assay (pLDH ELISA) was used to assess parasite growth inhibition after 72 h, following published methods [23]. pLDH optical densities (OD) were plotted against drug concentrations, and 50% inhibitory concentration (IC_50_) values were estimated by nonlinear regression analysis using GraphPad Prism 6.0 (GraphPad Software, Inc, San Diego, CA, USA).

For *P. falciparum* reference strains, parasites were maintained in culture using a modified Trager and Jensen method [24], and synchronized by 5% d-sorbitol [25]. Synchronized parasites with ≥ 90% ring forms were used for susceptibility testing as described by Chaorattanakawee et al. [22].

### Ring-stage survival assay (RSA) and piperaquine survival assay (PSA)

Sensitivity for dihydroartemisinin was assessed by a Ring-stage Survival Assay (RSA) and sensitivity to piperaquine (PPQ) using a piperaquine survival assay (PSA), following published methods [10, 26]. Briefly, parasites in in vitro culture were tightly synchronized using 5% d-sorbitol and 75% Percoll to obtain 0 to 3-h post‐invasion ring stage parasites. The synchronized parasite was adjusted to 1% parasitaemia, and cultured in a 48-well microplate with 700 nM DHA for 6 h (RSA) or 200 nM PPQ for 48 h (PSA). The culture medium was then discarded, cells were washed, re-suspended in a drug-free medium, and cultured for another 42 h (RSA) or 24 h (PSA). Susceptibility to DHA and PPQ was assessed microscopically on thin films by estimating the percentage of viable parasites, relative to drug-free controls (% survival rate). For quality control, the assays were also performed on *P. falciparum* reference clones, W2, 3D7, and C2B (ART-sensitive) and IPC-5202 (ART-resistance). ART and PPQ were kindly provided by the WWARN.

### *P. falciparum* cytochrome b mutation analysis for atovaquone resistance

*Plasmodium falciparum* cytochrome b (*pfcytb*) Y268S mutation was analyzed using polymerase chain reaction (PCR)-artificial Restriction Fragment Length Polymorphism (RFLP) genotyping. The nested PCR was performed to amplify the *pfcytb* fragment covering codon 268 using modified primers that could create the NsiI site (ATGCA/T) for wild type (268Y) and PstI site (CTGCA/G) for mutant 268S in PCR products. Primer sequences, annealing temperature, and size of PCR product are shown in Table 1. A 10 μL PCR reaction was carried out containing 0.5 μL of DNA template, 0.5 μM of each primer, 200 μM of mix dNTP, 0.25 units of Immolase DNA polymerase (Meridian Life Science Inc. USA), 1.5 mM MgCl_2_ in 10 mM Tris–HCl, pH8.3 and 50 mM KCl buffer. PCR product from the 1st round of PCR was diluted 1:50 with sterile distilled water and used as a DNA template for the 2nd round of PCR. PCR was performed using Perkin Elmer Gene Amp PCR System 9700 with hot start conditions as follows: first at 95 °C for 10 min followed by 30 cycles of 96 °C for 30 s, 55 °C or 58 °C for 30 s, 72 °C for 30 s, and at the end of 30 cycles, the extension was completed at 72 °C for 7 min. The PCR product was subsequently digested with NsiI-HF and PstI-HF (New England Biolabs, Inc., Ipswich, MA). The digestion reaction contained 2 μL of PCR product and 2 units of the restriction enzymes in the appropriate buffer. The reactions were incubated for 2 h at 37 °C followed by 20 min at 80 °C to inactivate the enzymes. The digestion reactions were analyzed on 1.5% agarose gel stained with RedSafe™ (iNtRON Biotechnology, Inc, Seongnam, Republic of Korea). Wild type (268Y) was defined as the digested band of 359 bp in NsiI reaction, but the non-digested 381 bp band in PstI reaction. On the other hand, mutant 268S was defined as the digested band of 359 bp in the PstI reaction, but non-digested band of 381 bp in NsiI reaction.

**Table 1:** Primer sequences, annealing temperature and the expected PCR product size

Primer name	5′-3′ sequences^*^	Tm	Size (bp) (bp)
PfCYTB33 (F)	ATT TAT GAT ATT TAT TGT AAC TGC	55 °C (1st round PCR)	565
PfCYTB4R (R)	AGT TGG TTA AAC TTC TTT GTT CTG C		
CytbY268S-F2 (F)	AGC AGT AAT TTG GAT ATG TGG AGG	58 °C (2nd round PCR)	381
CytB_268Y_RFLP (R) (R) (R)	TTA CTT GGA ACA GTT TTT AAC AAT GC		
CytB_268S_RFLP (R)	TTA CTT GGA ACA GTT TTT AAC ACT GC		

^*^Underlined bases indicate the mismatch base introduced in primers in order to generate the *Nsi*I site for wild-type parasite (268Y) and PstI site for mutant (268S) in PCR products. F: forward: R: reverse; Tm: annealing temperature

### Assessment of mutations in *P. falciparum Pfkelch13*

The whole length of the *Pfkelch13* gene (total 2181 bp, including one exon) was amplified by nested PCR and sequenced on an ABI Sequencer (Macrogen Inc, Seoul, Republic of Korea). Primer and PCR conditions were published previously [4]. To monitor cross contamination, negative control samples having no template were added to every PCR run. The results of sequencing were aligned to the *Pfkelch13* gene of reference strain 3D7 (potential 9PF13 0238 NCBI Reference Sequence (3D7): XM001350122.1). The bioinformatic analysis was conducted using Bioedit software. In case of polymorphic gene patterns, annotation was performed by two individuals blinded to the origin of the sample.

### Data analysis

Parasite drug susceptibilities and compound activity were expressed as median IC_50_ for all isolates and median IC_50_ of replicate data for each reference strain. Descriptive analysis was used to compare activity of rhinacanthin analogues 1 and 2 among field isolates and various reference strains. IC_50_ values to standard anti-malarials were compared to previously described IC_50_ resistance thresholds, which include: IC_50_ to CQ > 85 nM, IC_50_ to MQ > 24 nM, IC_50_ to QN > 351 nM, and ATQ > 1900 nM [27–29]. Although these thresholds established in the isotopic assay may not necessarily applicable to pLDH ELISA-based assay in the present study, they are the only one references well-defined for resistance. For parasite susceptibility to ART and PPQ, the % survival rate attained from RSA and PSA was calculated as described before [10, 26]. Published cut-off values denoting drug resistance are a survival rate > 1% and 10% for ART and PPQ resistance, respectively. Correlations in parasite susceptibility among rhinacanthin analogues and ATQ were analysed using Spearman’s correlation. Statistical analysis was performed using GraphPad Prism version 6.0 (GraphPad Software, Inc, San Diego, CA).

## Results

### Parasite susceptibility to rhinacanthin analogues and standard anti-malarials

**Fig. 2:**
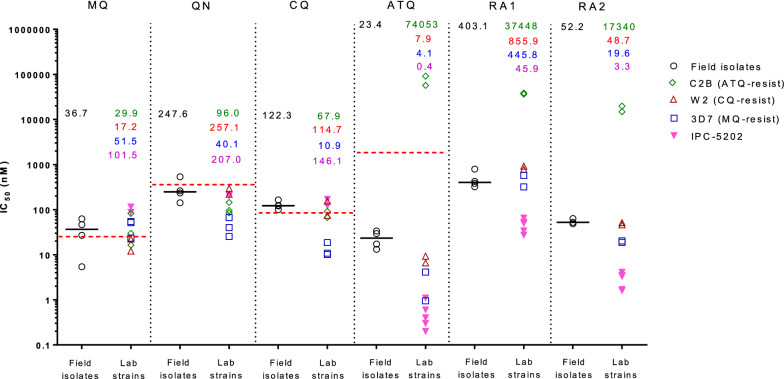
*Plasmodium falciparum* susceptibility to rhinacanthin analogues and standard anti-malarials. Scatter plots show IC_50_ to mefloquine (MQ), quinine (QN), chloroquine (CQ), atovaquone (ATQ), and rhinacanthin analogues 1 (RA1) and 2 (RA2) for 4 fresh field isolates (single experiment) and reference laboratory strains (2–5 replicate experiments). Field isolates and reference laboratory strains are colour-coded as in the legend, drug resistant profiles of reference strains were indicated in the legend. Bars represent the median IC_50_ values of field isolates, the median values of field isolates and reference strains are also indicated above the dot plot, accordingly. The red dashed lines indicate established resistance thresholds from the literature (IC50 to MQ > 24 nM, IC50 to QN > 351 nM, IC50 to CQ > 85 nM, and ATQ > 1,900 nM).

Four patient samples with parasitaemia ranging from 0.05 to 1.1% were tested for the in vitro susceptibility assay. Figure 2 shows the results of the parasite susceptibility assay to rhinacanthin analogues 1 and 2, and standard anti-malarials. More details on the susceptibility data are provided in Additional file 1. The assay on *P. falciparum* field isolates showed IC_50_s of 321.9–791.1 nM (median 403.1 nM), and 48.6–63.3 nM (median 52.2 nM) for rhinacanthin analogues 1 and 2, respectively. Similar results were obtained for the *P. falciparum* reference 3D7 and W2 strains. While the ART-resistant IPC-5202 strain was more sensitive to these compounds with a median IC_50_ of 49.5 nM and 3.3 nM for rhinacanthin analogues 1 and 2, respectively, the ATQ-resistant strain, C2B, showed high grade resistance (IC_50_ > 15,000 nM). Compared to ATQ, these 2 compounds were less potent against ATQ-sensitive parasites as shown by the higher IC_50_ value compared to ATQ, but more potent against ATQ-resistant parasites (C2B).

For the parasite susceptibility assay to standard anti-malarials, *P. falciparum* field isolates showed median IC_50_ values higher than the resistance threshold for mefloquine (MQ) and chloroquine (CQ), but lower than the threshold for quinine (QN) and ATQ. This finding suggested that *P. falciparum* isolates in this area are resistant to MQ and CQ, but remain sensitive to QN and ATQ. The reference laboratory strains showed susceptibility patterns conform to the known resistance profiles of the 3D7, W2 and C2B laboratory strains, resistant to MQ, CQ, and ATQ, respectively. The IPC-5205 strain, originating from western Cambodia, was shown to be resistant to both MQ and CQ.

### Ring-stage (RSA) and piperaquine survival assays (PSA)

**Fig. 3:**
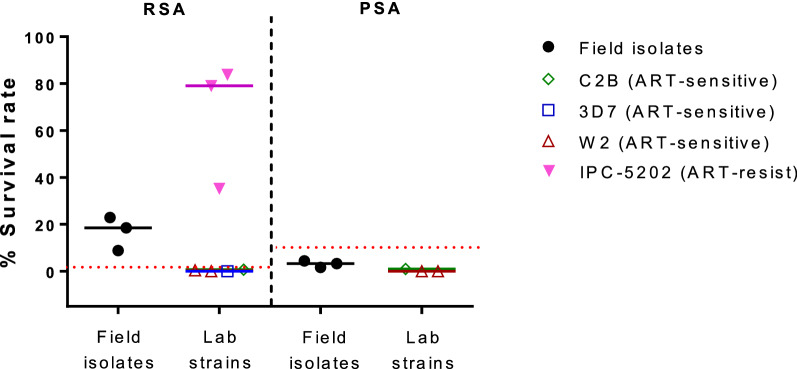
Ring-stage (RSA) and piperaquine survival assay (PSA) of *P. falciparum* field isolates and reference strains. Scatter plots show % survival rate of 3 culture-adapted field isolates as well as reference lab strains (1–3 replicate experiment). Field isolates and reference lab strains are color-coded as in the legend, artemisinins resistant profiles of reference strains were indicated. Bars represent the median values were indicated. The red dash lines indicate the resistance threshold for artemisinin and piperaqiune, RSA survival rates > 1% and PSA survival rates > 10%, respectively

Of the four *P. falciparum* field isolates included in drug susceptibility assays (NC001–NC004), 3 (NC002–NC004) were culture-adapted and tested in the RSA and PSA (Fig. 3). All evaluated isolates were resistant to ART, but remained sensitive to PPQ. All field isolates showed RSA survival rates > 1%, above the ART resistance cut-off, with a range of 8.8–22.9%. PSA survival rates were less than the 10% PPQ resistance cut-off with a range of 1.7–4.4%. The reference strains showed %RSA survival rates as expected of the known resistance patterns with a median of 79.1% for ART-resistant IPC-5202, and survival rates < 1% for ART-sensitive W2, 3D7, and C2B. W2, and C2B which are sensitive to PPQ showed a PSA survival rate ranging from 0–1% (Fig. 3). The dataset of RSA and PSA survival rate is available as Additional file 1.

### Susceptibility patterns of rhinacanthin analogues and atovaquone

Spearman correlation analysis was used to compare susceptibility patterns between rhinacanthin analogues 1, 2 and ATQ. There was a strong positive correlation between the in vitro activity of rhinacanthin analogues 1 and 2, and ATQ, expressed as IC_50_ (r = 0.83–0.97, P < 0.001).

### No cytochrome b Y268S mutation (ATQ resistance marker) were observed in *P. falciparum* field isolates

**Fig. 4:**
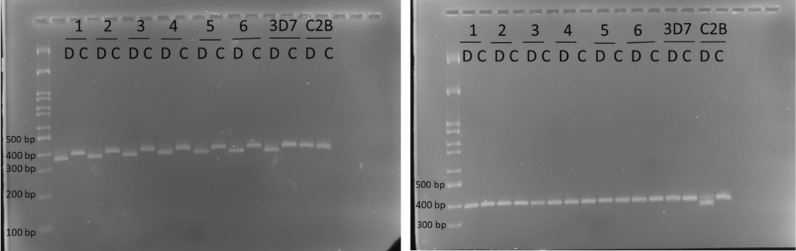
PCR-artificial Restriction Fragment Length Polymorphisms genotyping of *P. falciparum* cytochrome b Y268S mutation. Digestion reactions with *Nsi*I (left) and PstI (right) of 6 representatives of all field isolates (1–6), 3D7 and C2B strains are shown. Digestion reaction (D) and no digestion controls (C) were run in parallel. All isolates and the 3D7 strain were wild type (268Y) as indicated by the digested 359 bp band in the *Nsi*I reaction, but a non-digested 381 bp band in the PstI reaction. The C2B strain was confirmed to have a mutant 268S, defined as the digested band in the PstI reaction, but a non-digested band in the NsiI reaction.

Genotyping of the cytochrome b 268 position showed that all 4 *P. falciparum* field isolates included in the in vitro susceptibility study and other 100 parasite DNA samples from the same area were wild type (268Y) (Additional file 2). The same RFLP profile was observed for all evaluated isolates. The result of PCR–RFLP genotyping of 6 representative isolates is shown in Fig. 4. All *P. falciparum* isolates and 3D7 strains which were 268Y (wild type) showed the digested band of PCR products in the NsiI reaction, but not in PstI, while C2B strain which is 268S (mutant) showed the digested band in the PstI reaction, but not in NsiI. The result indicates that *P. falciparum* infections from this area remain sensitive to ATQ.

### Mutation frequency of *P. falciparum kelch13* gene

*Pfkelch13* propeller genotype of the four *P. falciparum* isolates included the in vitro susceptibility study and that of other 100 samples collected from the same province are presented in Additional file 2. Three of the four *P. falciparum* isolates tested for drug susceptibility exhibited the R539T mutation, whereas one sample was wild type. This corresponded with RSA results showing these 3 mutant isolates (NC002–NC004) exhibited survival rate above the ART resistance cut-off (> 1%), although RSA was not performed in the wild type isolate (NC001). For the other set of 100 samples from patients with uncomplicated falciparum malaria, there were point mutations in the propeller region of the gene in 91% (91/100) of the samples. C580Y was the predominant mutant type (70/100, or 70%), followed by R539T (19/100, or 19%). Two patient samples (2%) exhibited double mutations of C580Y and R539T, although it could not be excluded that this was explained by a multiclonal infection with both the C580Y and R539T mutant.

## Discussion

The present study is the first report evaluating in vitro activity of rhinacanthin analogues against multi-drug resistant *P. falciparum* isolates from northeast Thailand. These two rhinacanthin analogues 1 and 2, containing a naphthoquinone moiety resembling the corresponding structure in ATQ, has shown previously promising anti-malarial activity against a *P. falciparum* reference strain (K1). In the current study, the parasiticidal activity of two rhinacanthin analogues 1 and 2 against *P. falciparum* field isolates was shown. Analogue 2 was substantially more potent than analogue 1, with median IC_50_ of 52.2 nM against field isolates from an area of known multidrug resistance, while analogue 1 had an eightfold higher IC_50_ of 403 nM. Analogues 2 also had a similar profile against laboratory strains with known resistance patterns, including 3D7 (mefloquine-resistant), and W2 (chloroquine-resistant) strains, as well as the K1 strain tested in the previous study [16]. The ART-resistant strain IPC-5202 from western Cambodia had the highest sensitivity to these compounds, while the ATQ-resistant strain C2B had high-grade resistance. ATQ was more powerful against field isolates and laboratory strains, except for the ATQ-resistant strain, C2B that was more sensitive to these compounds than ATQ. The cross-susceptibility profile between rhinacanthin analogues and ATQ supports prior evidence of a similar mechanisms of action, selectively inhibiting the parasite cytochrome *bc*1 complex and mitochondrial function [16, 30, 31]. Rhinacanthin analogue 2 in particular is a strong candidate for further optimization, and could be developed as a new anti-malarial drug against multidrug resistant falciparum malaria. The main difference between analogue 1 and 2 is the longer lipophilic fatty acid side chain (14 carbons vs. 8) in analogue 2. As published previously, the longer side chain might confer increased anti-malarial activity by facilitating cell membrane penetration due to its higher lipophilicity [16].

All *P. falciparum* isolates included in this study were collected from Ubon Ratchathani province with several districts residing along the borders of southern Laos and northwest Cambodia. Parasite resistance to ART and partner drugs, and failure of ACT have been reported with increasing frequency [1, 9, 32, 33]. In vitro assay confirmed that the field isolates included in this study were resistant to MQ, CQ, and ART, but remained sensitive to ATQ and PPQ. The 2 rhinacanthin analogues demonstrated activity against these multi-drug resistant isolates. Analogue 2 had particularly potent activity against the IPC-5202 ART resistant strain with IC_50_ < 5 nM. Parasite drug susceptibility profiles from this study correspond to recent reports of parasite phenotypic and genotypic resistance from neighbouring provinces (Sisaket and Surin) across the Thai-Cambodian border. However, in vitro PPQ resistance among field isolates was observed in those regions with increased IC_50_ of 400 nM suggesting high-grade resistance, though a PSA was not done in that recent report [32]. Although Ubon Ratchathani province is adjacent to Sisaket and in close proximity to Surin, the present study collected parasite isolates from a district located at the Thai-Laos rather than the Thai-Cambodia border. The present isolates from the Thai-Laos border were more sensitive to PPQ than those from the Thai-Cambodia border, though the sample size is small, which is related to the sharp reduction in malaria transmission in this area. Also similar to these findings a multi-country clinical trial of DHA-PPQ treatment from 2015–2018 revealed very low treatment efficacy in Thailand (12.7%) and Cambodia (38.2%) along the Thai-Cambodia border, but 78% efficacy in northeast Cambodia along the Laos border [1, 14].

The GMS remains the epicentre of multi-drug resistant malaria, especially along Thai-Cambodian border. *Plasmodium falciparum* have adapted to resist to multiple previously effective drugs including CQ, sulfadoxine-pyrimethamine, MQ, and more recently ART and PPQ. Mutations or amplifications in drug resistance genes [34–36] have threatened the efficacy of ACTs which remain the most effective treatment for multi-drug resistant falciparum malaria. Most recently, DHA-PPQ failure was detected soon after its adoption in Thailand, Cambodia, and Vietnam [8, 14, 37]. This was associated with the rapid spread of drug resistant parasites. Molecular analysis in the GMS between 2013 and 2018 revealed significant increases in ART resistance (the *kelch13* C580Y) and PPQ resistance markers (*plasmepsin 2/3* amplifications, as well as CQ resistance transporter mutations) [1, 32, 38, 39]. Parasite genomic studies revealed a strong selective pressure on the ART and PPQ-resistant co-lineages, resulting in aggressive resistance expansion [40, 41]. New, more effective regimens are desperately needed.

Parasite population genetics have evolved dynamically as a result of rapid changes in drug regimens, parasite fitness, intensive human migration and parasite population flow across regional borders. To date, genetic evolution relating to ART and ACT partner drugs has been the most widely reported [40, 41]. ATQ is another anti-malarial with a different mode of action, targeting the cytochrome bc1 complex [29, 42]. It has been used in limited areas with high-grade ACT failure in combination with proguanil (PG) as an ACT alternative [43, 44]. For the moment, the use of ATQ-PG has been relatively sparse, and clinical resistance is minimal, even in the GMS. In the present study, *P. falciparum* cytochrome b (*pfcytb*) genotyping of a hundred isolates from various districts of Ubon Ratchathani province bordering Cambodia and Laos collected in 2014–2015 did not detect any mutations in *pfcytb*. The lack of relevant genetic evidence and in vitro susceptibility data confirmed that isolates in this area remain sensitive to ATQ. This support earlier reports from northern Cambodia from 2013 to 2015, and Thailand from 1988 to 2015 where all isolates tested were sensitive to ATQ and did not carry the genetic marker for ATQ resistance [12, 32, 44, 45]. Clinical trials of Malarone® (ATQ-PG) in Cambodia from 2009 to 015 [46, 47] and Thailand 1998–2002 [43, 48] also showed acceptable efficacy of 90–93% and 98–100% 28-day cure rates, respectively. Therefore, rhinacanthin analogue 2, which shows very similar resistance patterns as ATQ, seem to be an attractive candidate for further development for use in areas with artemisinin and ACT partner drug resistance. However, similar to ATQ, a single mutation on amino acid residue 268 of *pfcytb* could confer high grade resistance to these compounds, raising concern on rapid selection of the *pfcytb* mutation conferring resistance. To date, selection of the *pfcytb* mutation has not occurred, probably because drug pressure from atovaquone-proguanil (ATQ-PG) has been low, although ATQ-PG has been used in some areas along Thai-Cambodia border as part of ACT resistance containment activity since 2009 [12, 44, 47]. For further development of the rhinacanthin analogues, further lead optimization will be necessary to improve the parasiticidal activity of rhinacanthin analogues 1 and 2. The basic structure of naphthoquinone reveals that four functional groups play significant roles: (i) the 20 substituents of the propyl chain, (ii) the chain length of the aliphatic chain, (iii) the R-methyl substituent of the aliphatic chain, and (iv) the ester group [16, 49]. The synthesis of modified anti-malarial naphthoquinones is being studied, which could aid in the development of future anti-malarial drugs caused by mitochondria-containing parasites. In addition, the development of nanocarriers for targeted delivery to enhance mitochondrial penetration may increase anti-malarial activity [50]. Alterations to modify actions on the cytochrome bc1 complex may improve activity against *pfcytb* mutant isolates.

## Conclusions

Two rhinacanthin analogues 1 and 2 showed parasiticidal activity against multi-drug resistant *P. falciparum* isolates, but analogue 2 was considerably more potent. An artemisinin-resistant laboratory strain IPC-5202 originating from western Cambodia was most sensitive to the rhinacanthin analogues 1 and 2, respectively, whereas the atovaquone-resistant laboratory strain C2B had high-grade resistance to both compounds. The cross-susceptibility profile between rhinacanthin analogues and atovaquone was confirmed. Further optimization of the rhinacanthin analogues is required to increase activity and ideally to improve efficiency in killing atovaquone-resistant isolates. Rhinacanthin analogues could be a useful new drug class for the treatment of multi-drug resistant falciparum malaria.

## Supplementary Information

**Additional file 1. **IC_50_ to rhinacanthin analogues 1 and 2, and standard anti-malarials, and % Ring-stage (RSA) and Piperaquine Survival rate (PSA) of *P. falciparum* field isolates and reference strains. Data attained from 1 to 5 replicate experiments are presented. NA. indicates data not available as no experiment was done.

**Additional file 2. ***P. falciparum Cytochrome b* Y268S and *Kelch13* propeller genotype of the four *P. falciparum* isolates included in the in vitro drug assay (NC001–NC004) and other 100 samples collected from the same province (THA001-100).

## Data Availability

The dataset supporting the conclusions of this article is included within the article and its additional files.

## Abbreviations

ACT: Artemisinin based combination therapy
AS: Artesunate
ATQ: Atovaquone
ART: Artemisinins
CQ: Chloroquine
DHA: Dihydroartemisinin
ELISA: Enzyme-linked immunosorbent assay
IC_50_: 50% Inhibitory concentration
MQ: Mefloquine
OD: Optical density
PCR: Polymerase chain reaction
*pfcytb*: *P. falciparum* Cytochrome b
PG: Proguanil
pLDH: *Plasmodium* Pan-species lactate dehydrogenase
PPQ: Piperaquine
PSA: Piperaquine survival assay
QN: Quinine
RA: Rhinacanthin analogue
RFLP: Restriction Fragment Length Polymorphisms
RSA: Ring-stage survival assay
WWARN: World-Wide Antimalarial Resistance Network
